# Randomized clinical trial of the effect of the onset time of skin-to-skin contact at birth, immediate compared to early, on the duration of breastfeeding in full term newborns

**DOI:** 10.1186/s13006-021-00379-z

**Published:** 2021-04-13

**Authors:** Sergio I Agudelo, Oscar A Gamboa, Eduardo Acuña, Lina Aguirre, Sarah Bastidas, Jennifer Guijarro, María Jaller, María Valderrama, María Lucia Padrón, Nathalie Gualdrón, Evelyn Obando, Fabio Rodríguez, Lina Buitrago

**Affiliations:** 1 Facultad de Medicina, Universidad de La Sabana, Chía, Colombia; 2 Departamento de Pediatría, Hospital Universitario de La Samaritana – Unidad Funcional de Zipaquirá, Zipaquirá, Colombia

**Keywords:** Infant, Newborn, Skin-to-skin contact, Breastfeeding, Neonatal care, Kangaroo mother care

## Abstract

**Background:**

Skin-to-skin contact (SSC) compared to separation at birth has a positive effect on breastfeeding. However, separation at birth is common with negative impact on breastfeeding. The aim was to determine the effect of immediate SSC compared to early SSC on the duration of exclusive breastfeeding.

**Methods:**

A randomized multicentre parallel clinical trial was conducted in two hospitals in Cundinamarca (Colombia) between November 2018 and January 2020. Low-risk full term newborns at birth were included. Neonates were assigned to immediate (in the first minute after birth) or early onset (start exactly at 60 min of life) skin to skin contact. Monthly follow-up was performed until 6 months of age. The primary outcome was the percentage of exclusively breastfed infants at 6 months (time in months with human milk as the only source of food). Secondary outcomes were the percentage of infants with exclusive breastfeeding at 3 months, duration in months of exclusive breastfeeding, neonate’s breastfeeding ability, percentage of weight change between birth and the first week of life and hospitalization in the neonatal unit in the first week. A bivariate analysis was performed to determine the variables associated with exclusive breastfeeding at 6 months. A survival analysis was performed to evaluate the effect of the onset of SSC on exclusive breastfeeding duration.

**Results:**

A total of 297 newborns were included: 49.8% (*n* = 148) in the immediate SSC group, and 50.2% (*n* = 149) in the early SSC group. The mean duration of exclusive breastfeeding in both groups was 5 months. There were no differences between the groups in the percentage of exclusive breastfeeding at 6 months (relative risk [RR] 1.06, 95% CI 0.72, 1.58) or in the duration of exclusive breastfeeding (hazard ratio [HR] 0.98, 95% CI 0.74, 1.28).

**Conclusions:**

The percentage of infants and the duration of exclusive breastfeeding in the first 6 months of age were the same between the two groups of SSC interventions. Given the current barriers to its implementation, the results of this study could positively impact the use of SSC at birth and standardize the intervention and improve breastfeeding indicators.

**Trial registration:**

ClinicalTrials.gov NCT02687685.

## Background

Skin-to-skin contact (SSC) at birth between mother and child consists of placing the naked newborn in the prone position on the naked thorax of the mother between her breasts at the time of birth [1]. It is part of the set of essential newborn care interventions that has a positive impact on the health of the mother and newborn [2, 3]. In fact, SSC, compared with mother-child separation at birth, improves breastfeeding indicators (early onset, prevalence and duration of exclusive breastfeeding), physiological stability during immediate adaptation, risk of hospitalization prior to discharge from the maternity ward and the mother-child bond and attachment [4, 5].

The main effect of SSC on the health of newborns and infants is derived from the consequences of breastfeeding [6–8]. Exclusive breastfeeding in the first 6 months of life is a strategy that reduces infant morbidity and mortality [9, 10]. However, the prevalence of exclusive breastfeeding in the world is 37% [9], and in Colombia, 36.1% of children under 6 months received exclusive breastfeeding [11]. Increasing the prevalence of exclusive breastfeeding is a priority in all nations, with the goal of increasing it by at least 50% by 2025 [12].

The prevalence of SSC in the care of the mother-infant dyad during birth is very variable among different regions of the world, with its prevalence being much lower than ideal [13, 14]. The situation is similar in Colombia, where mother-child separation is frequent [15, 16]. Some identified barriers to achieving its broad and general use in the care of the mother-child dyads are the perception by health professionals of interfering with the care routines of the mother and child at birth, limited time availability and personnel to carry out the intervention, lack of knowledge of the technique and its benefits, fear of complications and absence of guidelines and standardization of the practice [17–19].

The time of onset of SSC can be categorized as immediate, beginning in the first 10 min after birth, and as early, beginning in the first hour of life [1, 4]. However, the time of onset is very heterogeneous in studies on SSC, making it difficult to interpret the results and standardize the intervention [4, 13]. Moreover, there are no studies that compare the effect of onset in the first 10 min versus in the first hour [4, 13]. It is thus necessary to standardize the technique in regard to the time of onset and duration [13].

Given the positive effects of SSC, expanding its use in the care of the mother-child dyads would contribute to the objectives of improving the prevalence of exclusive breastfeeding and reducing neonatal and infant mortality. Knowing the existing barriers and the lack of standardization of the procedure, evaluating the two onset times of SSC and their effects on neonatal health and breastfeeding could help overcome such barriers and improve the evidence-based guidelines that recommend the best technique for performing the intervention while maintaining its effects on health. The objective of this study was to determine the effect of immediate SSC compared to early SSC on the duration of exclusive breastfeeding in full term and healthy newborns. The hypothesis that there is a difference in the percentage of full term newborns who receive exclusive breastfeeding for 3 months or more between the immediate and early SSC at birth groups was evaluated. The study protocol was registered in the clinicaltrials.gov clinical trials database under identification number NCT02687685, and the study protocol has been previously published [20].

## Methods

### Study design

A randomized multicentre parallel clinical trial was conducted in two second-level reference hospitals in the Sabana Centro Cundinamarca region (Hospital El Salvador de Ubaté and Hospital Universitario de La Samaritana – Unidad Funcional de Zipaquirá) between November 2018 and January 2020.

### Ethical consideration

The study protocol was approved by the ethics committee of Universidad de La Sabana (N° 70–2018) and Hospital Universitario de La Samaritana (N° 0302–18). The parents of the neonates signed an informed consent for inclusion in the study prior to the start of and/or in the latent phase of labour and before randomization. There was a data monitoring and security committee headquartered at the Contract Research Organization (CRO). The researchers were planned an interim analysis from the protocol when completing 50% of the sample size (150 newborns) followed up to 3 months.

### Study population

Full term newborns with appropriate weight for gestational age, born from single gestation and by vaginal birth, with spontaneous neonatal adaptation, stable at birth, with indication for maternity and whose mother expressed desire to breastfeed were included. Neonates with congenital malformations, mothers with perinatal complications, patients with indication for separation of the mother-child pair at the time of birth and mother-child dyads with known contraindications for breastfeeding were excluded.

### Outcomes

The primary outcome was the percentage of neonates with exclusive breastfeeding at 6 months. Secondary outcomes were the percentage of infants with exclusive breastfeeding at 3 months, duration in months of exclusive breastfeeding, neonate’s breastfeeding ability evaluated by the Infant Breastfeeding Assessment Tool (IBFAT) in the first 24 h and/or before discharge and categorized into effective vigorous breastfeeding (IBFAT score ≥ 10) and non-effective breastfeeding (moderately effective and weak sucking and/or no feeding; IBFAT score < 10) [21, 22], percentage of weight change between birth and the first week of life (less than or equal to 7% and greater than 7%), and hospitalization in the neonatal unit in the first week.

For the primary outcome, a survey was applied at the follow-ups with the neonates developed according to WHO recommendations for breastfeeding practices [23]. The type of foods and supplements given to the infant during the past month was asked, categorizing the feeding practice into [24]: exclusive breastfeeding, predominant breastfeeding, partial breastfeeding and complementary feeding. Last, based on this information, the results were categorized into exclusive breastfeeding and non-exclusive breastfeeding. Exclusive breastfeeding was considered the time in months with human milk as the only source of food with no other liquids or solid foods given [23].

For the application of the IBFAT, prior to the start of recruitment an educational strategy and a pilot test were performed to standardize the use of the instrument. The weight change in the first week was evaluated in the face-to-face follow-up in the first week of life, and it was measured on the same electronic scale used at birth. The need for hospitalization was investigated at this same follow-up; when the mother reported hospitalization, the event was reviewed and documented with the medical record.

### Randomization and blinding

The mother-child dyads were randomly allocated to one of two study groups, immediate SSC and early SSC, through a computer-generated process centralized in the CRO and with a strategy of permuted blocks with size of six. The allocation numbers were kept hidden in opaque and sealed envelopes; they were only consecutively revealed in the final expulsion phase of labour by the study monitor assigned to the centre. Due to the characteristics of the study intervention, blinding was only applied to the group of researchers who analysed the data.

### Intervention

SSC between mother and child was defined as placing the naked newborn in the prone position, wearing a diaper and cap, on the mother’s naked thorax (between the breasts), with both covered by a warm blanket [1]; the duration of SSC in both groups was established as 1 h (60 min).

Immediate SSC was defined as the intervention initiated in the first minute after birth, with performance of the immediate neonatal adaptation manoeuvres (thermoregulation, umbilical cord clamping and immediate neonate assessments) during SSC but postponing subsequent manoeuvres (neonate evaluations and screenings) until the end of the SSC duration. Early SSC was defined as the intervention that began exactly at 60 min of life of the newborn. In this group, thermoregulation and umbilical cord clamping were performed according to the protocol of the health institution, after which the neonate was placed under a radiant warmer to complete the neonatal adaptation interventions; once these were completed, the neonate was dressed and placed next to the mother, initiating the SSC at 60 min of life. In both study groups, during the SSC, the neonate was monitored with a pulse oximeter, three-lead EKG, axillary temperature measurement and continuous medical surveillance, recording these data at the beginning and end of the intervention. Mothers were advised and supported to initiate early breastfeeding (first hour of life) in both groups.

The neonates were followed-up for a total period of 6 months. The first follow-up was performed at discharge from the maternity ward, when the IBFAT score was evaluated. The second follow-up was performed in the first week of life (between five to 7 days of life) in person in the doctor’s office. The weight and need for hospitalization during the first week of life were recorded. Subsequently, monthly follow-ups were conducted by telephone by applying the survey to evaluate the practice of breastfeeding.

### Statistical analysis

The sample size was calculated considering a baseline risk of exclusive breastfeeding at 6 months for Colombia of 24% [25], and considering that we found no studies evaluating the two onset times of SSC, the data in Moore et al. were used [26], who reported that SSC compared to separation at birth increases exclusive breastfeeding between three and 6 months of age (relative risk [RR] 1.97, 95% CI 1.37, 2.83), for a power of 80% and a Type I error probability of 5% with expected losses to follow-up of 30%. Thus, a total sample size of 300 neonates was estimated (150 in each group).

Descriptive data analyses were performed using measures of central tendency, position and dispersion for the continuous variables and absolute and relative frequencies for the categorical variables. The assumption of normality was evaluated with the Shapiro-Wilk test. The association between the time of onset of SSC and categorical variables (exclusive breastfeeding at six and 3 months, percentage of weight change, hospitalization in the first week of life and newborn’s breastfeeding behaviours) were evaluated by constructing contingency tables and evaluating the independence between variables with the Chi^2^ test and/or Fisher’s exact test according to the assumption of number of cases per cell. The RR was calculated as a measure of effect along with its 95% confidence interval (95% CI).

A survival analysis was performed to evaluate whether the time of onset of SSC has an effect on the duration of exclusive breastfeeding. For the analysis, the final event was the absence of exclusive breastfeeding. The event time was defined from the time of onset of the intervention until the month that exclusive breastfeeding was documented. In participants who were lost to follow-up, this time was censored until the last follow-up in which exclusive breastfeeding was documented. Survival functions were constructed using the non-parametric Kaplan-Meier method for the total cohort and by SSC onset time group. Survival functions were compared using the log-rank test. A Cox proportional hazards analysis was performed to estimate the hazard ratio (HR) with the 95% CI of exclusive breastfeeding suspension. Subgroup analyses were also performed for variables considered to possibly interact with the evaluated interventions (early breastfeeding, newborn breastfeeding behaviours, hospitalization in the first week of life and receiving breastfeeding education at discharge from the maternity ward). The proportionality assumption was confirmed by Cox regression models.

For data collection, input and management, the electronic data capture tool REDCap (Research Electronic Data Capture) housed at the Institute for the Evaluation of Health Care Quality (Instituto para la Evaluación de la Calidad y Atención en Salud - IECAS) was used. Data analysis was performed in STATA version 14. Because an interim analysis was planned from the protocol, the *p -* value was adjusted for multiple analyses, defining a value of *p* < 0.025 as the level of significance.

## Results

**Fig. 1:**
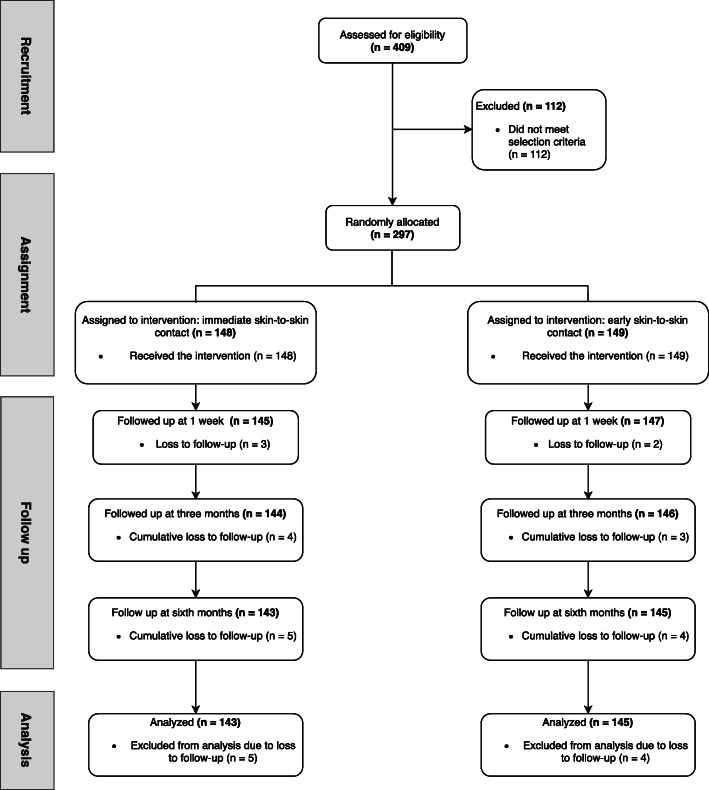
Flowchart

A total of 297 newborns were included, 148 (49.8%) in the immediate SSC group and 149 (50.2%) in the early SSC group (Fig. 1). There was loss to follow-up for the primary outcome of exclusive breastfeeding at 6 months of nine participants, five in the immediate SSC group and four in the early SSC group, with no differences in baseline characteristics.

The two study groups were similar, especially regarding confounding variables such as maternal obesity, working and parity (Table 1). No differences were observed between the groups in the percentage of exclusive breastfeeding at 6 months (SSC immediate 27.3% vs SSC early 25.5%; RR 1.06, 95% CI 0.72, 1.58) or at 3 months (SSC immediate 79.9% vs SSC early 80.1%; RR 0.99, 95% CI 0.88, 1.11).

**Table 1:** Sociodemographic and clinical characteristics of the mother-child dyads by group skin-to-skin contact

	Immediate skin-to-skin contact (***n*** = 148)	Early skin-to-skin contact (***n*** = 149)
Maternal Characteristics		
Age, years (median - IQR)	23 (21–29)	24 (20–25)
Marital status *n* (%)		
Domestic partnership	112 (75.7)	109 (73.2)
Separated/divorced	0	1 (0.67)
Married	14 (9.5)	11 (7.4)
Single	22 (14.9)	28 (18.8)
Educational level *n* (%)		
Primary education	12 (8.1)	19 (12.7)
Secondary education	95 (64.2)	85 (57.0)
Higher education	16 (10.8)	20 (13.4)
Graduate education	2 (1.3)	1 (0.67)
Technical, professional and/or technological education	22 (14.9)	17 (11.4)
University or equivalent	1 (0.68)	7 (4.7)
Specialization	0	0
Doctorate	0	0
Working mother *n* (%)		
Yes	107 (72.3)	109 (73.1)
No	41 (27.7)	40 (26.8)
Socio-economic stratum *n* (%)		
1	91 (61.5)	100 (67.1)
2	53 (35.8)	47 (31.5)
3	2 (1.3)	2 (1.34)
4	1 (0.68)	
5	1 (0.68)	
Primiparous *n* (%)		
Yes	79 (53.4)	81 (54.4)
No	69 (46 .6)	68 (45.6)
Previous breastfeeding *n* (%)		
Yes	75 (94.9)	77 (95.1)
No	4 (5.1)	4 (4.9)
Smoking *n* (%)		
Yes	1 (0.68)	1 (0.67)
No	147 (99.3)	148 (99.3)
Antenatal appointments, median (IQR)	8 (6–9)	7 (5–9)
BMI classification at the beginning of pregnancy *n* (%)		
Low weight	4 (2.7)	10 (6.7)
Normal	105 (70.9)	95 (63.8)
Overweight	26 (17.6)	32 (21.5)
Obesity	13 (8.8)	12 (8.0)
Newborn characteristics		
Birthweight (grams) median (IQR)	3085 (2805–3340)	3130 (2920–3400)
Gestational age (Weeks - Ballard) median (IQR)	39 (38–40)	39 (38–39)
Sex *n* (%)		
Male	66 (44.6)	71 (47.6)
Female	82 (55.4)	78 (52.3)

Immediate skin-to-skin contact: intervention initiated in the first minute after birth

Early skin-to-skin contact: intervention that began exactly at 60 min of life of the newborn

Similarly, no differences were found in the breastfeeding behaviours of the newborns at discharge from the maternity ward (IBFAT score), hospitalization in the first week of life and/or the weight change percentage between birth and the first week of life (Table 2).

**Table 2:** Time of onset of skin-to-skin contact and health outcomes

	Immediate skin-to-skin contact ***n*** (%)	Early skin-to-skin contact ***n*** (%)	RR^**a**^ (95% CI)
Exclusive breastfeeding^b^ to 6 months			1.06 (0.72, 1.58)
Yes	39 (27.3)	37 (25.5)	
No	104 (72.7)	108 (74.5)	
Exclusive breastfeeding^b^ to 3 months			0.99 (0.88, 1.11)
Yes	115 (79.9)	117 (80.1)	
No	29 (20.1)	29 (19.9)	
IBFAT^c^ score at discharge			1.02 (0.94, 1.10)
Effective	134 (90.5)	132 (88.6)	
Not Effective	14 (9.7)	17 (11.4)	
Weight loss during the first week of life			0.82 (0.45, 1.49)
> 7%	17 (11.7)	21 (24.3)	
≤ 7%	128 (88.9)	126 (85.7)	
Hospitalization in the first week of life			0.6 (0.22, 1.63)
Yes	6 (4.1)	10 (6.8)	
No	139 (95.7)	137 (93.2)	

^a^RR = Relative risk comparing immediate SSC versus early SSC

^b^Exclusive breastfeeding: was considered the time in months with human milk as the only source of food with no other liquids or solid foods given

^c^*IBFAT* Instrument Breastfeeding Assessment Tool

**Fig. 2:**
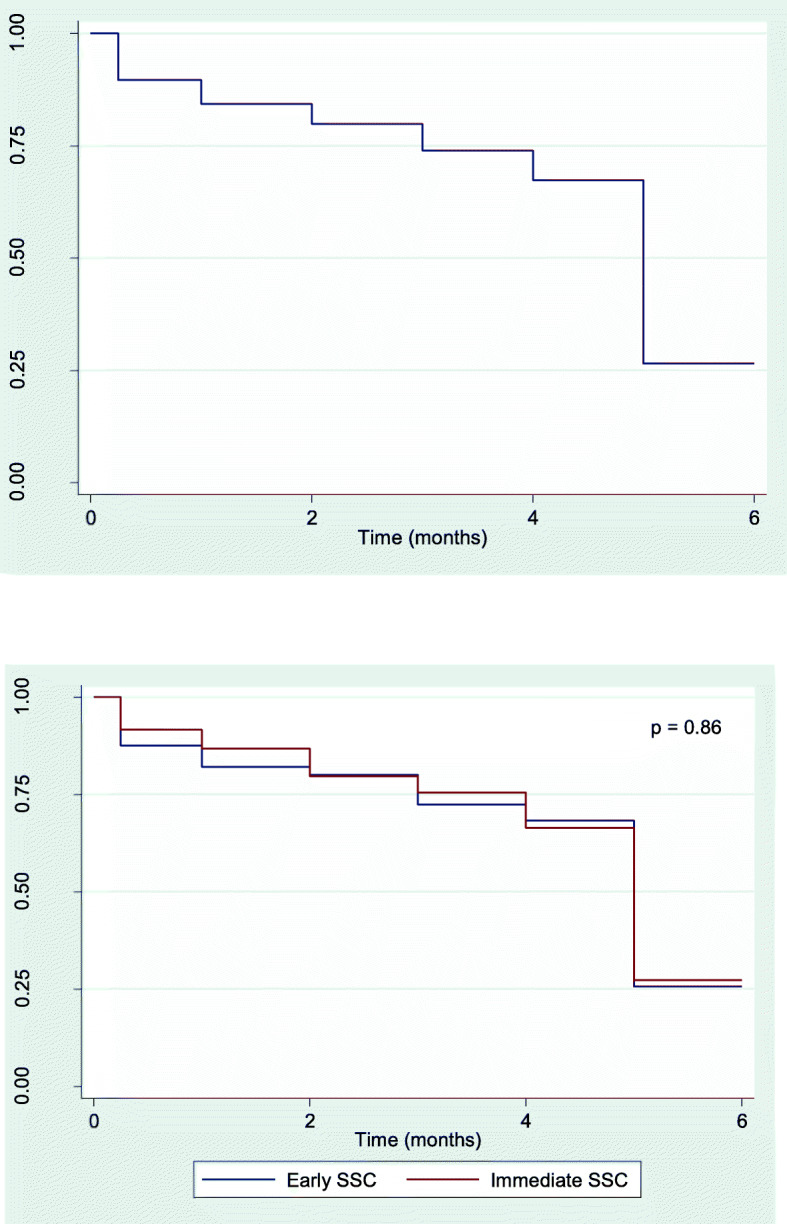
Kaplan-Meier plot of exclusive breastfeeding. **a** - Kaplan-Meier plot of exclusive breastfeeding for the total cohort. **b** - Kaplan-Meier plot of exclusive breastfeeding for the cohort by group. *p = Long-rank test*. SSC = Skin-to-skin contact

**Fig. 3:**
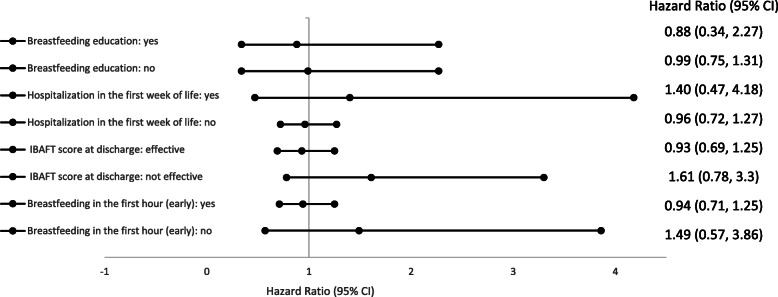
Subgroup survival analyses

Figure 2a and b shows the survival function for the total cohort and for the SSC onset time groups, respectively. The median exclusive breastfeeding duration was 5 months for the total cohort and for both groups. The Cox proportional hazards model showed no differences in the duration of exclusive breastfeeding between the two study groups (HR 0.98, 95% CI 0.74, 1.28). In the subgroup analysis (breastfeeding education, hospitalization, IBFAT score, breastfeeding in the first hour), no differences were observed in exclusive breastfeeding duration (Fig. 3).

## Discussion

Our trial evaluated the effect of the time of onset of SSC, immediate versus early, on exclusive breastfeeding in the first 6 months of age. The results indicate that placing low risk newborns in SSC for one continuous hour immediately after birth (in the first minute after birth) and/or after performing routine newborn and maternal care practices (at 60 min of life) is the same in the two SSC study groups in the percentage of infants on exclusive breastfeeding at the three and 6 months of life and in the duration of exclusive breastfeeding. Similarly, no differences were observed between the groups in relation to the behaviour of newborns towards breastfeeding, weight loss and hospitalization in the first week.

Compared to separation at birth, SSC is effective in different areas of mother-child health, especially in improving indicators and prevalence of breastfeeding in infants under 6 months [27, 28]. Specifically, it increases the percentage of infants on exclusive breastfeeding at 3 months and 6 months. Karimi et al. [28] showed an effect of SSC started in the first 10 min of life compared to separation in improving the duration of exclusive breastfeeding for 3 months (odds ratio [OR] 2.47, 95% CI 1.73, 3.48) and for 6 months (OR 1.71, 95% CI 1.05, 2.78). Likewise, Moore et al. [4] showed that SSC started immediately compared to separation increases the duration of breastfeeding by 3 months or more (RR 1.97, 95% CI 1.37, 2.83). However, there was a question about the effect of the onset of SSC on breastfeeding and the need to standardize the strategy to improve its recommendation and applicability [4, 13].

In the study, the median duration of exclusive breastfeeding in both groups was 5 months and the rates of exclusive breastfeeding in the trial were higher than in rates reported for the country. Where the main decrease in exclusive breastfeeding is observed in the first month of life, where 51.6% of infants receive exclusive breastfeeding [11] and the percentage of infants with exclusive breastfeeding in our trial was 89.6% (immediate SSC 87.6% and early SSC 91.6%). The duration of exclusive breastfeeding in Colombia is approximately 2 months [25]. Our trial show that in a group of low-risk neonates, SSC, regardless of the time of onset, improves the percentage of exclusively breastfed infants and is an effective intervention that aims at increasing the number of neonates exclusively breastfed in the first 6 months by 50%.

The results of the present study are relevant since the barriers to performing SSC are the perception of interference with the newborn care routines and the availability of time and personnel during birth and immediate neonatal adaptation [17, 19]. The strategy of initiating SSC once the mother and child are stabilized and the newborn interventions are finished, could positively impact the use of SSC at birth without compromising exclusive breastfeeding.

The results of the present study also show no differences between the time of onset of SSC groups in hospitalization in the first week of life or in the newborn behaviours evaluated by the IBFAT. Again, this is important given that previous studies have shown that separation at birth compared to SSC negatively impacts these parameters. Agudelo et al. [5] reported that SSC initiated in the first 10 min of life and maintained for 45 continuous minutes decreased the risk of hospitalization prior to discharge from the maternity ward (13.8% vs. 26.4%; OR 0.46, 95% CI 0.29, 0.71). Khadivzadeh et al. [29] found that the success of the first breastfeeding evaluated by the IBFAT was significantly higher in the group with SSC immediately after birth (first 10 min) compared to the group that adapted under a radiant warmer. Moore et al. [4] showed that the group exposed to SSC compared to separation had a higher IBFAT score (mean difference 2.28, 95% CI 1.41, 3.15).

Some of the main risk factors for non-exclusive breastfeeding are maternal obesity, working and smoking [30–33]. In addition, for Colombia, one of the main causes of non-exclusive breastfeeding is a sick/hospitalized child and maternal return to work [11]. We did not find differences in the duration of exclusive breastfeeding in the subgroups of obese, working and smoking mothers and neonate hospitalization in the first week of life. It is important to emphasize that during the study, in both interventions, mothers were encouraged and supported to perform early onset (first hour of life) breastfeeding and educational support was provided to all mothers.

A small loss to follow-up percentage and 100% adherence to the interventions and follow-ups established are strengths of this study. One of its limitations are the lack of a separation group at birth. However, we do not consider it feasible from an ethical point of view to have this group since offering SSC at birth is the currently recommended management for the care of the mother-child dyads and not offering it has ethical limitations.

## Conclusions

The percentage of infants and the duration of exclusive breastfeeding in the first 6 months of age were the same between the two groups of SSC interventions. Given the low prevalence of the use of SSC at birth and the current barriers to its implementation, the results of the present study may contribute to increase the prevalence of the use of SSC and to standardize the intervention by supporting the development of guidelines for the technique. Increasing the prevalence of Skin-to-Skin Contact would improve exclusive breastfeeding.

## Data Availability

The datasets generated and/or analyzed during the current study are available in the electronic data capture tool REDCap (Research Electronic Data Capture) housed at the Institute for the Evaluation of Health Care Quality (Instituto para la Evaluación de la Calidad y Atención en Salud - IECAS). The datasets generated are not publicly available due the confidentiality and information of the participating minors requested by the health institutions but are available from the corresponding author on reasonable request.

## Abbreviations

CRO: Contract Research Organization
IBFAT: Infant Breastfeeding Assessment Tool
REDCap: Research Electronic Data Capture
SSC: Skin-to-Skin Contact
